# Müllerian duct anomalies: review of current management

**DOI:** 10.1590/S1516-31802009000200007

**Published:** 2009-07-06

**Authors:** Sérgio Conti Ribeiro, Renata Assef Tormena, Thais Villela Peterson, Marina de Oliveira Gonzáles, Priscila Gonçalves Serrano, José Alcione Macedo de Almeida, Edmund Chada Baracat

**Affiliations:** 1 MD, PhD. Head of Laparoscopic Surgery Group, Department of Gynecology and Obstetrics, Faculdade de Medicina da Universidade de São Paulo (FMUSP), São Paulo, Brazil.; 2 MD. Attending physician, Laparoscopic Surgery Group, Department of Gynecology and Obstetrics, Faculdade de Medicina da Universidade de São Paulo (FMUSP), São Paulo, Brazil.; 3 Medical student, Faculdade de Medicina da Universidade de São Paulo (FMUSP), São Paulo, Brazil.; 4 MD, PhD. Head of Pediatric and Adolescent Group, Department of Gynecology and Obstetrics, Faculdade de Medicina da Universidade de São Paulo (FMUSP), São Paulo, Brazil.; 5 MD, PhD. Chief professor, Department of Gynecology and Obstetrics, Faculdade de Medicina da Universidade de São Paulo (FMUSP), São Paulo, Brazil.

**Keywords:** Urogenital abnormalities, Müllerian ducts, Embryonic development, Laparoscopy, Infertility, Gynecology., Anormalidades urogenitais, Ductos de Müller, Desenvolvimento embrionário, Laparoscopia, Infertilidade, Ginecologia.

## Abstract

The aim of this paper was to discuss the embryological aspects of Müllerian duct anomalies and to analyze the current diagnostic methods and therapy. Müllerian anomalies are congenital defects of the female reproductive tract resulting from failure in the development of the Müllerian ducts and their associated structures. Their cause has yet to be fully clarified, and it is currently believed to be multifactorial. Symptoms appear principally during adolescence or early adulthood, and affect the reproductive capacity of these women. When clinically suspected, investigations leading to diagnosis include imaging methods such as hysterosalpingography, ultrasonography and magnetic resonance. The classification of these malformations relates to their embryogenesis, and defines the therapy and prognosis. Müllerian anomalies consist of a wide range of defects that may vary from patient to patient. Therefore, their management must also be individual, taking anatomical and clinical characteristics into consideration, as well as the patient’s wishes.

## RESUMO

O objetivo deste trabalho foi discutir as malformações müllerianas desde seus aspectos embriológicos, analisando os atuais métodos diagnóstico e terapêuticos. As malformações müllerianas são anomalias congênitas do trato reprodutivo feminino decorrentes de falha do desenvolvimento dos ductos de Müller e estruturas associadas. Sua causa não foi completamente elucidada, acreditando-se, atualmente, que seja multifatorial. Os sintomas se manifestam, principalmente, durante a adolescência e início da vida adulta, e afetam a capacidade reprodutiva dessas mulheres. A partir da suspeita clínica, a investigação diagnóstica inclui métodos de imagem, como a histerosalpingografia, ultrassonografia e ressonância magnética. A classificação das malformações está relacionada à sua embriogênese e direciona a terapêutica e prognóstico. As malformações müllerianas são um grupo amplo de anomalias que variam de paciente para paciente. Portanto, sua abordagem também é individual, devendo-se considerar os aspectos anatômicos, clínicos e o desejo da paciente.

## INTRODUCTION

Müllerian duct anomalies consist of a set of structural malformations resulting from abnormal development of the paramesonephric or Müllerian ducts. The prevalence of these anomalies ranges from 0.001 to 10% in the general population and from 8-10% in women with an adverse reproductive history.^1^^,^^2^ The embryological development of the female reproductive system is closely related to the development of the urinary system, and anomalies in both systems may occur in up to 25% of these patients.^3^ Other associated malformations may affect the gastrointestinal tract (12%) or musculoskeletal system (10-12%).^3^^,^^4^

Gonad formation begins between the fifth and sixth weeks of pregnancy, with the appearance of the urogenital ridge developing from the intermediate mesoderm and the migration of the germinative cells originating in the coelomic epithelium. Female development is determined by the absence of the Y chromosome (and consequent absence of the factor that determines testicle development) and by the presence of two X chromosomes.^3^^,^^4^

At around the ninth week, the ovaries are formed and the Wolffian (mesonephric) and Müllerian ducts coexist. Absence of testosterone leads to involution of the Wolffian duct, whereas absence of anti-Müllerian hormone allows differentiation of the Müllerian duct. The caudal portions of these ducts merge to form the uterovaginal canal, which later gives rise to the cervix and uterus, as well as to the upper third of the vagina. Complete development of the vagina occurs through fusion of the structures of the urogenital sinus and the Müllerian tubercle. The cranial portion of the duct, i.e. the part that does not fuse, opens into the peritoneal cavity, giving rise to the Fallopian tubes.^3^^,^^4^

The causes of Müllerian anomalies have yet to be fully clarified. The karyotypes are normal (46 XX) in 92% of the women with Müllerian anomalies and abnormal (sex chromosome mosaicism) in 8% of these women. The majority of these developmental abnormalities are infrequent and sporadic, and are thus attributed to polygenic and multifactorial causes.^5^ A recent study attributed persistence of the intrauterine septum to a deficiency in the antiapoptotic protein Bcl 2, which is responsible for the process of apoptosis and absorption of the septum.^5^

Events such as hypoxia that occur during pregnancy, the use of medications such as methotrexate or diethylstilbestrol (DES), ionizing radiation and viral infections may also contribute towards the occurrence of Müllerian malformations.^6^^,^^7^ Among the drugs that induce Müllerian malformations, thalidomide and DES lead to malformations of the uterine cavity. DES, a nonsteroidal estrogen, was widely used in the 1950s, principally in the United States, for treating various obstetrical conditions, particularly miscarriage and preeclampsia. Consequently, anomalies consisting notably of vaginal adenocarcinoma and deformations of the uterine cavity (specifically, T-shaped uterus) were identified in the daughters of women who had taken this medication.^8^ A study carried out on rats exposed intrauterinely to DES reported modifications to Dkk2, Nkd2, sFRP1, hox, Wnt and Eph gene expression. These have been correlated with changes induced by this drug.^9^

In a study on cases of congenital uterine and vaginal agenesis (Mayer-Rokitansky-Küster-Hauser syndrome), possible mutations and polymorphisms were evaluated in the anti-Müllerian hormone gene and in the gene for its receptor. Analysis of the hox genes (the genes relating to embryogenesis) and the N314D gene (the gene that codifies the enzyme galactose-1-phosphate uridyl transferase [GALT] for galactose metabolism) failed to find any positive correlation with the presence of malformations.^10^^,^^11^

The aim of this study was to discuss embryology, diagnostic methods and therapy in cases of Müllerian duct anomalies. We searched PubMed (National Library of Medicine, Bethesda, Maryland, United States) by using the following terms: “Müllerian Ducts”[Mesh] OR “Urogenital Abnormalities”[Mesh]. The search strategy is described in Table 1.

**Table 1.** Database search results

Database	Strategy	Result
PubMed*	“Müllerian Ducts”[Mesh] OR “Urogenital Abnormalities”[Mesh]	161 clinical trials 43 randomized controlled trials 4 meta-analyses 1451 case reports 8 practice guidelines
Lilacs (Literatura Latino-Americana e do Caribe em Ciências da Saúde)		9 clinical trials 46 case reports 25 reviews 1 practice guideline 1 epidemiological study
Cochrane		22 clinical trials

*Limits: added to PubMed in the last 10 years; Female; English; Portuguese.

## PHYSIOPATHOLOGY

Uterine malformations result from failure in organogenesis or from fusion or reabsorption of the Müllerian ducts. Failures in organogenesis are related to incomplete development of one or both Müllerian ducts, thereby leading to agenesis, uterine hypoplasia or a unicornuate uterus.^3^

Fusion defects result from incomplete merging of the caudal portion with the Müllerian ducts (lateral fusion) or incomplete merging of the structures of the urogenital sinus with the Müllerian tubercle (vertical fusion). Failures in lateral fusion may result in uterus didelphys, bicornuate uterus or arcuate uterus. When the defect occurs in vertical fusion, anomalies such as imperforate hymen, transverse vaginal septum, oblique vaginal septum or absence of the cervix may result. Following caudal fusion of the ducts, the remaining portion of the central septum is reabsorbed. Reabsorption failure results in a uterus with a partial or complete septum. Certain malformations of the uterine cavity also lead to the formation of hypoplastic uterus, infantile uterus, agenesis of the cervix and T-shaped uterus.^3^

## CLASSIFICATION

The definition of uterine anomalies proposed by the American Fertility Society^12^ classifies uterine malformations into seven separate categories:

Class I: Hypoplasia/uterine agenesis.

Class II: Unicornuate uterus:

- Has a functioning endometrium and communication with the main uterine cavity.
- Also has an endometrial structure that responds to hormonal stimulus; however, there is no communication with the external genital tract.
- Has a rudimentary structure with no activity, attached to a more fully developed uterine horn.
- Results from the development of only one Müllerian duct, with complete agenesis of the contralateral duct.

Class III: Uterus didelphys.

Class IV: Bicornuate uterus:

- Complete: when the indentation produced in the fundic region is deep, thus indicating that fusion failed from the level of the cervical region.
- Incomplete: when the division is higher, not extending to the level of the cervix, indicated by the shallower indentation in the contour of the region of the uterine fundus.

Class V: Septate uterus:

- When the septum extends into the internal cervical ostium, possibly including the cervical canal, and divides the cervix into two tunneled cavities. A vaginal septum is often also present.
- When the septum does not divide the uterine cavity along its entire length, and circulation exists between the two chambers.

Class VI: Arcuate uterus:

This is a rather insignificant anomaly of the uterine cavity in which, generally, no abnormalities in the external contour of the uterus are visible. The small fundal cleft or impression with a protruding uterine horn becomes more noticeable during pregnancy.

Class VII: T-shaped uterus resulting from the use of DES.

## CLINICAL IMPLICATIONS

Müllerian anomalies are frequently asymptomatic and are often missed in routine gynecological examinations. Nevertheless, a history of pelvic pain following the menarche, dysmenorrhea and an increase in abdominal volume are complaints suggestive of uterine anomalies. In addition, primary amenorrhea and changes to menstrual flows may be present.^13^

Among the ductal differentiation malformations, vaginal agenesis presents with primary amenorrhea and dyspareunia. In cases of uteri with a functional endometrium, hematometra and hematocolpos are frequent findings.

A unicornuate uterus is seldom symptomatic unless associated with other malformations. If a rudimentary, noncommunicating uterine horn is present together with a functional endometrium, hematometra and sometimes hematosalpinges may be found.^14^

The clinical presentation of anomalies associated with defects in fusion and reabsorption of the septum varies clinically according to the duct segment affected. The presence of a vaginal septum is perceived by patients as an obstacle to sexual relations and may be confirmed by speculum examination. When the menstrual flow is obstructed, patients may report pain.^12^

Uterine septum is generally an asymptomatic condition and is often only diagnosed when couples with a history of repeated miscarriage or infertility are undergoing investigation. Likewise, lateral fusion defects, which are responsible for uterus didelphys and bicornuate uterus, are often detected only when women undergo imaging tests.^12^

Anomalies resulting from failure in vertical fusion, such as cervical agenesis, transverse vaginal septum and imperforated hymen, are associated with primary amenorrhea, hematocolpos and hematometra.^15^

The reproductive prognosis for patients with Müllerian malformations is shown in Table 2.^3^^,^^12^^,^^16^^,^^17^^,^^18^^,^^19^ The hypotheses developed to explain the poor obstetrical prognosis for women with the diverse Müllerian malformations include decreased intraluminal volume; inadequate vascularization of regions such as the septum; presence of a medial wall or an unfused uterine horn; and greater uterine contractility and irritability, thereby leading to miscarriages and premature deliveries.^12^

**Table 2.** Reproductive prognosis

	Miscarriage (%)	Premature delivery (%)	Fetal survival (%)
Septate uterus^3^^,^^14^^,^^16^	> 60	-	6-28
Unicornuate uterus^17^	43.8	25	43.7
Uterus didelphys^11^	35	19	60
Bicornuate uterus	40^17^	-	62^17^

## DIAGNOSIS

Due to the complexity of presentations, diagnosing of Müllerian malformations requires the use of more than one imaging method in 62% of the cases.^20^^,^^21^ Hysterosalpingography (HSG) is the method traditionally used to evaluate the cervical canal, uterine cavity and Fallopian tubes. Its efficacy in diagnosing anomalies is debatable and varies according to the specific type of malformation.^20^ The specificity values range from 6 to 60%, depending on the malformation investigated and the technician’s skill.^1^^,^^22^ HSG enables accurate evaluation of tube permeability and can detect the presence of uterine septa, intrauterine synechiae, submucous fibroids and endometrial polyps. However, it cannot be performed on patients who are virgins and it does not allow the external uterine anatomy to be viewed, which hampers the differential diagnosis between uterus didelphys and septate uterus.^3^^,^^22^^,^^23^ Moreover, the method exposes the patient to ionizing radiation; the injection of contrast may cause allergies and discomfort; and there is also a risk of uterine perforation and infection.^1^

Ultrasonography has a sensitivity of around 44%, varying according to the specific type of malformation under evaluation, the patient’s body composition, the radiologist’s experience and the type of transducer used. Transvaginal ultrasonography allows a more detailed analysis of the endometrium, uterine cavity and cervix. The specificity of this examination ranges from 85 to 92%.^24^^,^^25^^,^^26^ Recently, three-dimensional ultrasonography has shown high specificity and sensitivity in evaluations on all uterine anomalies, including Müllerian malformation.^27^

The specificity of magnetic resonance imaging (MRI) ranges from 96 to 100% for diagnosing Müllerian malformations.^1^ In cases of uterine hypoplasia, the images show a small endometrial cavity and a shorter distance between the uterine horns.^20^ In these patients, the presence of ovaries and associated renal malformations can also be evaluated. In cases of unicornuate uterus, a small endometrial cavity with only one Fallopian tube is seen. A rudimentary uterine horn may be present and, if there is no communication with the cervical canal, the accumulated retrograde menstruation will be easily seen.^20^

Uterus didelphys is visible in axial sections, which show two separate chambers. However, the anatomy of the wall is intact. A vaginal septum may or may not be present.^20^

The MRI criteria for diagnosing a bicornuate uterus consist of the presence of divergent uterine horns and concavity at the contour of the uterine fundus. This type of anomaly appears as a heart-shaped uterus. On the other hand, in cases of septate uterus, the external contour of the uterus is normal and the septum is seen as a difference in signal intensity, according to its composition. Fibrous septa are seen as low-intensity signals on T2-weighted images and muscular septa as intermediate-intensity T2 signals.^28^ Nowadays, cytogenetic analysis on patients with developmental anomalies of the Müllerian ducts may only be useful for family counseling.^29^

## THERAPEUTIC MANAGEMENT

The treatment for Müllerian anomalies varies according to the specific type of malformation found in each patient. A systematic search (descriptor: Müllerian anomalies or uterine anomalies, default tag: Title/Abstract) was conducted in the Lilacs, PubMed and Cochrane databases. Two randomized controlled trials were identified.^30^^,^^31^ Most of the studies on therapeutic issues (827 studies) were restricted to reports on single cases or small series.

Anomalies of the vaginal septum should be resected at the time of diagnosis, thereby resolving problems of dyspareunia and permitting adequate drainage of menstrual flow.^32^

Patients with a confirmed diagnosis of cervical agenesis should be referred for hysterectomy, preferentially performed laparoscopically. Several surgical attempts to create a cervix have resulted in tragic outcomes, often associated with fatal complications.^19^ The prospects for pregnancy using *in vitro* fertilization techniques should be evaluated in the light of the obstetrical complications, and possible alternatives should be offered to these women. The use of a surrogate womb may be the best option in such cases. In cases of uterine septum, the resection should be performed hysteroscopically, in order to improve the reproductive prognosis for these patients by decreasing the incidence of miscarriage, premature delivery and infertility.^17^ The advantages of hysteroscopy include the shorter duration of surgery, smaller blood loss, lower costs, reduced morbidity and shorter hospital stay, compared with abdominal surgery.^30^^,^^31^

In cases of complete uterine septum, resection of the cervical septum may be related to cervical incompetence and secondary infertility. A randomized controlled trial performed to evaluate the safety and efficacy of resection of the cervical septum during hysteroscopic metroplasty showed that this procedure was safer and easier with resection than with preservation of the cervical septum.^32^

The use of estrogen therapy or an intrauterine device are postsurgical alternatives for minimizing formation of uterine adherences (synechiae).^32^^,^^33^ The follow-up in these cases includes hysteroscopy, one to three months after the initial surgery.^31^ Laparoscopy should be used to excise obstructed, rudimentary uterine horns and adjacent tubes in patients with a unicornuate uterus. It should also be used for hysterectomy in cases of cervical agenesis and in neovaginoplasty procedures in cases of vaginal agenesis.^19^ In many women, the malformation results in obstructed and retrograde menstruation, thereby facilitating the development of endometriosis. During laparoscopy, this diagnosis may be confirmed and the endometrial foci may be resected.

## CONCLUSIONS

Müllerian anomalies consist of a wide range of defects that may vary from patient to patient. Therefore, their management must also be individual, taking anatomical and clinical characteristics into consideration, as well as the patient’s wishes.
